# Controlled Delivery of Pan-PAD-Inhibitor Cl-Amidine Using Poly(3-Hydroxybutyrate) Microspheres

**DOI:** 10.3390/ijms222312852

**Published:** 2021-11-27

**Authors:** Dina Ahmed, Hima Puthussery, Pooja Basnett, Jonathan C. Knowles, Sigrun Lange, Ipsita Roy

**Affiliations:** 1 Tissue Architecture and Regeneration Research Group, School of Life Sciences, University of Westminster, London W1W 6XH, UK; w1614534@gmail.com; 2 School of Life Sciences, University of Westminster, London W1W 6XH, UK; himapadman@gmail.com (H.P.); p.basnett@westminster.ac.uk (P.B.); 3 Department of Biomaterials and Tissue Engineering, Eastman Dental Institute, University College London, Royal Free Hospital, Rowland Hill Street, London NW3 2PF, UK; j.knowles@ucl.ac.uk; 4 Department of Materials Science and Engineering, Faculty of Engineering, University of Sheffield, Sheffield S10 2TN, UK

**Keywords:** peptidylarginine deiminase inhibitor, Cl-amidine, Poly(3-Hydroxybutyrate) microspheres, encapsulation, controlled drug delivery

## Abstract

This study deals with the process of optimization and synthesis of Poly(3-hydroxybutyrate) microspheres with encapsulated Cl-amidine. Cl-amidine is an inhibitor of peptidylarginine deiminases (PADs), a group of calcium-dependent enzymes, which play critical roles in a number of pathologies, including autoimmune and neurodegenerative diseases, as well as cancer. While Cl-amidine application has been assessed in a number of in vitro and in vivo models; methods of controlled release delivery remain to be investigated. P(3HB) microspheres have proven to be an effective delivery system for several compounds applied in antimicrobial, wound healing, cancer, and cardiovascular and regenerative disease models. In the current study, P(3HB) microspheres with encapsulated Cl-amidine were produced in a size ranging from ~4–5 µm and characterized for surface morphology, porosity, hydrophobicity and protein adsorption, in comparison with empty P(3HB) microspheres. Cl-amidine encapsulation in P(3HB) microspheres was optimized, and these were found to be less hydrophobic, compared with the empty microspheres, and subsequently adsorbed a lower amount of protein on their surface. The release kinetics of Cl-amidine from the microspheres were assessed in vitro and expressed as a function of encapsulation efficiency. There was a burst release of ~50% Cl-amidine in the first 24 h and a zero order release from that point up to 16 days, at which time point ~93% of the drug had been released. As Cl-amidine has been associated with anti-cancer effects, the Cl-amidine encapsulated microspheres were assessed for the inhibition of vascular endothelial growth factor (VEGF) expression in the mammalian breast cancer cell line SK-BR-3, including in the presence of the anti-proliferative drug rapamycin. The cytotoxicity of the combinatorial effect of rapamycin with Cl-amidine encapsulated P(3HB) microspheres was found to be 3.5% more effective within a 24 h period. The cells treated with Cl-amidine encapsulated microspheres alone, were found to have 36.5% reduction in VEGF expression when compared with untreated SK-BR-3 cells. This indicates that controlled release of Cl-amidine from P(3HB) microspheres may be effective in anti-cancer treatment, including in synergy with chemotherapeutic agents. Using controlled drug-delivery of Cl-amidine encapsulated in Poly(3-hydroxybutyrate) microspheres may be a promising novel strategy for application in PAD-associated pathologies.

## 1. Introduction

Peptidylarginine deiminases (PADs) constitute a family of calcium-dependent enzymes, with five isoenzymes in mammals that are PAD1, 2, 3, 4 and PAD6. PADs induce the post-translational deimination of arginine residues to produce citrulline in a calcium dependent manner, leading to structural and functional changes in target proteins [1,2,3]. The different PAD isozymes display tissue-specific expression and preferences for specific target proteins, although some are overlapping [4]. The PAD isozymes that have hitherto gained most interest in relation to autoimmune pathologies, neurodegeneration and cancer are PAD2 and PAD4 [2,4,5], whilst PAD3 has also gained considerable attention in relation to CNS regeneration [6,7] as well as in CNS related cancers [8,9]. Both PAD2 and PAD4 are linked to a range of cancers [3,9,10,11], and PAD3 has furthermore recently been highlighted in aggressive brain and pancreatic cancers [8,9,10]. PAD2 is the most broadly expressed isoform in tissues, including in the CNS, and is involved in CNS plasticity and transcription regulation, whilst PAD4 is mainly localized in the white blood cells and epithelial cells and is involved in chromatin decondensation, transcription regulation, tumorigenesis and innate immunity [2,11,12,13].

In relation to the CNS, both PAD2 and PAD4 are activated during development, also due to calcium oscillations, but are not normally activated in adults [11,14], whilst PAD3 has furthermore been associated with neuronal stem cells [15] and CNS regeneration [6,7]. However, both PAD2 and 4 have been found to play crucial roles in the development of immune-mediated and/or neurodegenerative diseases due to calcium dysregulation and the detection of increase in deiminated proteins in the CNS is associated with numerous neurodegenerative diseases such as multiple sclerosis (MS), Alzheimer’s disease (AD) and Parkinson’s disease (PD) [16,17,18,19,20].

One of the main consequences of post-translational deimination/citrullination mediated by PADs in the CNS was initially the observation of roles in multiple sclerosis (MS) due to the initiation and progression of autoimmune responses against the myelin sheath [18]. This is triggered due to neo-epitope exposure [21], leading to the development of this most common chronic, neurodegenerative, autoimmune disease. Furthermore, in various studies carried out using preclinical animal models of MS; transgenic mice with multiple copies of cDNA that encodes PAD2 [22] and mice with MOG-induced-experimental autoimmune encephalomyelitis (EAE) [23], it was shown that the rapid progression of MS is mainly due to PAD-mediated citrullination of myelin basic protein, which is the main component of myelin sheath [2,24,25]. PAD2 overexpression accompanied with deiminated proteins was also detected in the hippocampal region of Alzheimer’s patients [26,27], and furthermore, increased protein deimination has been observed in pre-motor PD brains [19].

Roles for PADs in acute CNS injury have also been described, and PAD inhibition using the pan-PAD inhibitor Cl-amidine was shown to effectively reduce brain injury following hypoxic ischemic insult (HI) in a murine model [7,28]. Roles for PADs in CNS regeneration were also demonstrated, showing that PAD3 contributed to the modulation of secondary injury response following spinal cord damage and that Cl-amidine could effectively promote CNS regeneration [6]. The pan-PAD inhibitor, Cl-amidine, [29,30] is well established and has already been assessed in a number of in vitro and in vivo models, including autoimmune diseases, cancer models and CNS neuroprotection with potent neuroprotective effects [3,6,7,8,20,31,32,33,34,35]. In previous studies of breast cancer models, Cl-amidine was reported to have anticancer effects either alone or in combination with other anti-mitotic chemotherapeutic agents such as docetaxel [34,35]. The reported anticancer effects of Cl-amidine administration involved the induction of cell cycle arrest and apoptosis through the enhancement of p53 nuclear accumulation, which regulates the expression of the OKL38 gene, a mediator of apoptosis [35,36]. In addition, Cl-amidine co-administration with chemotherapeutic agents enhances their effects through promoting the sensitization of cancer cells via extracellular vesicle (EV) modulation [34]. Furthermore, both Cl-amidine and PAD-isozyme specific inhibitors have been found to be effective in other in vitro cancer cell models such as glioblastoma and pancreatic cancer [8,9,10]. As many of the above mentioned studies used single administration of Cl-amidine following experimental injury, there is further need to develop methods and understanding for application of controlled Cl-amidine delivery for application in disease models. This would for example be important in relation to treatment in autoimmune diseases, progressive neurodegenerative diseases, CNS damage and regeneration, as well as in cancer.

Therefore, the generation of a tailored delivery system for PAD inhibitors, including Cl-amidine, is warranted. In the current study, an attempt to develop targeted delivery approaches for Cl-amidine took advantage of using the polyhydroxyalkanoate (PHA) biopolymers, which are already used and were previously validated in regenerative studies [37,38,39,40,41,42,43]. Furthermore, a proposed anti-cancerous effect of Cl-amidine was assessed, upon its co-administration with rapamycin, through investigating its effect on VEGF expression in a mammalian breast cancer cell line (SK-BR-3). VEGF has been extensively reported as a pivotal promoter of cancer cells metastasis through mediating angiogenesis [44,45,46,47,48,49]. VEGF has furthermore been attributed as a key player in CNS injury and regeneration [50,51,52].

Currently, drug encapsulation into polymeric micro- and nano-particles is the most commonly used drug delivery method. Micro/nano-particle encapsulation provides controlled and sustained drug release, which warrants an efficient pharmacological response. Among the well-established production techniques of micro/nanoparticles are coacervation, spray drying and numerous emulsion techniques [53]. Besides, various supercritical fluids-based techniques have further been developed to overcome the inconsistency within the produced particle sizes along with other drawbacks that could be associated with the conventional production methods [54,55,56]. In these techniques, carbon dioxide (scCO_2_) is the most successfully used supercritical fluid that can be applied either for solvent or, more commonly, for antisolvent co-precipitation of the polymeric carrier along with the drug as fine powders [57,58]. The supercritical fluid-based co-precipitation technique, in addition to the type of polymeric carrier used, produces a bimodal release pattern of the encapsulated drug [54,59,60]. The release pattern is characterized by an initial burst release for immediate symptoms alleviation, followed by a sustained release which is the pattern of choice for chronic conditions. This antisolvent co-precipitation technique was not applied within this study due to the insolubility of the solute drug, Cl-amidine, within the organic solvent, chloroform. 

In this current study, PHA biopolymers were used [61]. Initially, PHA biopolymers are produced intracellularly by certain bacterial species through fermentation in the form of a storage material. Consequently, these polymers are purified, extracted and remodelled into either micro or nanoparticles by applying an emulsion methodology [62]. The family of Polyhydroxyalkanoates (PHAs) is widely used in medical applications and is currently considered as one of the most promising group of biopolymers due to being highly biocompatible, bioresorbable, non-cytotoxic and non-carcinogenic [63]. This high safety profile favours the PHAs over other biodegradable polymers such as: polylactic acid (PLA) and poly(lactic-co-glycolic acid) (PLGA) which produce acidic by-products that provoke immune responses besides rendering the patients prone to toxicity [63]. PHAs are water insoluble linear polyesters of 3, 4, 5 and 6-hydroxyacids, synthesized by various microorganisms, through fermentation using limited nitrogen sources and excess carbon [38]. 

Both Poly(3-hydroxyoctanoate), P(3HO) and Poly(3-hydroxybutyrate), P(3HB), which are produced by *Pseudomonas mendocina* and the modified *Bacillus subtilis* OK2 strain respectively, are members of PHAs which are able to exhibit high efficacy when used in regenerative studies [64,65,66,67,68,69,70,71]. Both P(3HO) and P(3HB) exhibit outstanding outcomes when used as supporting scaffolds in cardiac, nerve, bone and skin tissue engineering [69,70,72,73,74]. In addition, P(3HB) has been proven to provide a well-tailored vehicle for drug delivery when used to formulate drug encapsulated microspheres, both in terms of encapsulation efficiency and drug release [39,40,75]. 

Thus, for novel drug-development, Cl-amidine was used as an exemplar PAD inhibitor in the current study for assessment of effectivity of PAD-inhibitor encapsulation in a controlled drug-delivery system comprising P(3HB) microspheres, which has previously been established as a safe and effective delivery method in various disease models. This novel drug delivery system may offer a sustained and tailored release rate according to the disease treated and provide longer term, safe release of the pan-PAD inhibitor Cl-amidine in a range of chronic disorders and cancer.

## 2. Results

### 2.1. P(3HB) Microsphere Optimization and Characterization

Cl-amidine was encapsulated in P(3HB) microspheres according to previously established methods [75], and the process was further optimized to obtain smaller microspheres of size ranging between 4–5 µm and maximum encapsulation efficiency according to the conditions presented in Table 1. Cl-amidine encapsulated P(3HB) microspheres corresponding to condition 3 (Table 1) were used for further characterization since this was the optimum condition producing the desired microsphere diameter and maximum encapsulation efficiency, compared with the preceding conditions. The results of these were then compared with empty microspheres which were used as control, in addition to rhodamine encapsulated P(3HB) microspheres which were used as another type of control due to the hydrophilicity of rhodamine [76,77] beside its fluorescent nature that could be used for future in vivo work, as discussed in Section 4.2.

### 2.2. Surface Morphology of the Cl-Amidine Encapsulated P(3HB) Microspheres Using SEM

**Figure 1:**
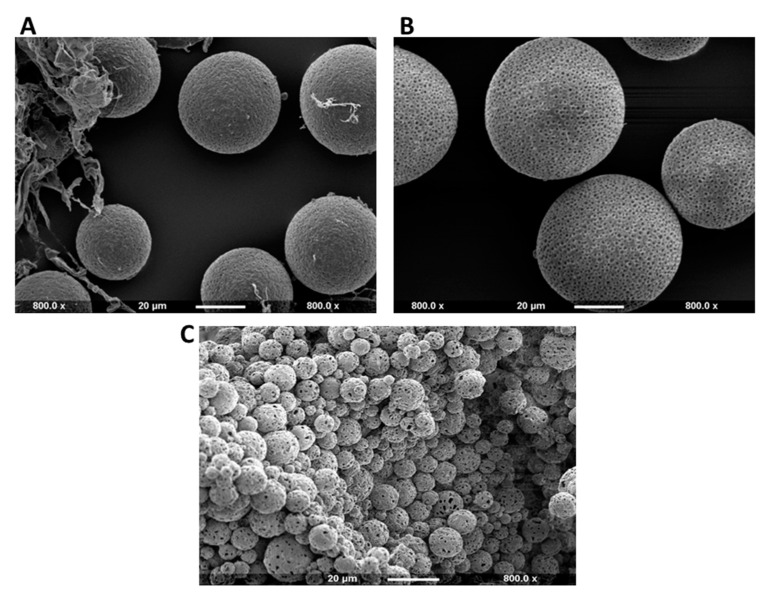
SEM images showing the surface morphology of Cl-amidine encapsulated P(3HB) microspheres produced under three different fabrication conditions. (**A**) Representative image of polydisperse microspheres and microsphere flakes produced under condition 1 (average diameter = 40 µm). (**B**) Representative image of polydisperse microspheres produced under condition 2 (average diameter = 50–60 µm); (**C**) Representative image showing the desired monodispersed P(3HB) microspheres with Cl-amidine encapsulated produced under condition 3 (average diameter = 4–5 µm). The scale bar represents 20 µm in all figures, and all images are taken at 800.0× magnification. The size of the scale bar was set as a reference to compare the Cl-amidine containing P(3HB) microsphere sizes produced under the 3 different conditions.

**Figure 2:**
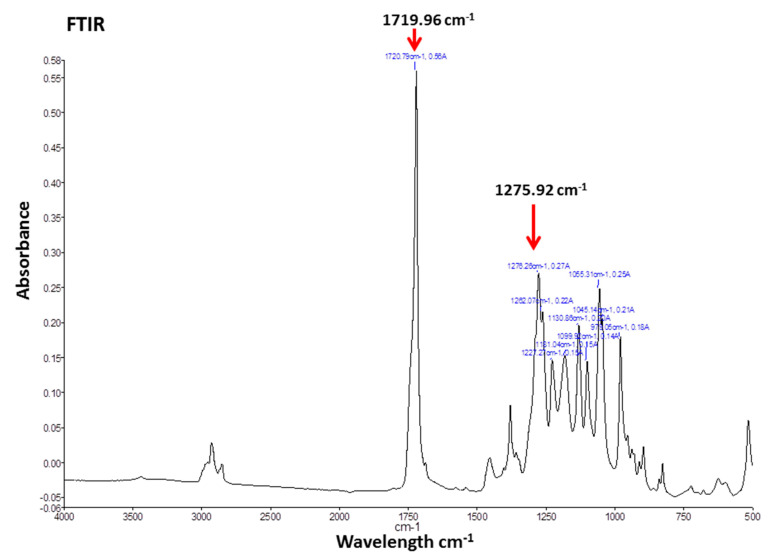
FTIR of Cl-amidine encapsulated P(3HB) microspheres. The FTIR spectra of Cl-amidine encapsulated P(3HB) microspheres exhibiting two distinct peaks at wave numbers of 1719.96 and 1275.92 cm^−1^, which correspond to the ester carbonyl and methyl groups, respectively, both of which are characteristic of the P(3HB) biopolymer used with which the microspheres are produced.

The SEM images obtained from the production of P(3HB) microspheres with encapsulated Cl-amidine, according to the different conditions presented in Table 1, are shown in Figure 1A–C. The surface morphology of the microspheres produced under the three conditions appears to be uniform as most of the particles are spherical in shape and exhibit porosity. Nevertheless, some polymeric flakes are present, mainly in condition 1 (Figure 1A). As for the particle size polydispersity, both conditions 1 (Figure 1A) and 2 (Figure 1B) showed a noticeable degree of polydispersity among the majority of the microspheres produced, whilst the majority of microspheres produced under condition 3 (Figure 1C) were monodisperse. Both conditions 1 (Figure 1A) and 2 (Figure 1B) produced relatively large sized microspheres with diameters of 40 and 50–60 µm, respectively. Conversely, P(3HB) microspheres produced under condition 3 were within the desired diameter range of 4–5 µm. This relatively low diameter range indicates increased surface area-to-volume ratio of the microspheres which directly increases the rate of encapsulated drug release. Thus, condition 3 was considered as the most optimal one and was further used in the continued experiments.

### 2.3. Fourier Transform Infrared Spectroscopy (FTIR) of Cl-Amidine Encapsulated P(3HB) Microspheres

The FTIR spectra of Cl-amidine encapsulated P(3HB) microspheres provided two distinct peaks at a similar range of wavelengths as those produced by P(3HB), indicating the absence of impurities (Figure 2). The FTIR spectra obtained with the microspheres exhibited two distinct peaks at wave numbers of 1719.96 cm^−1^ corresponding to the ester carbonyl group and at 1275.92 cm^−1^ corresponding to the methyl group (Figure 2), both of which are characteristic of the small-chain-length polyhdroxyalkanoates (SCL-PHAs), to which the P(3HB) belongs [78]. Supplementary Figure S1 furthermore shows an overlay of FTIR spectra of both empty and P(3HB) microspheres with Cl-amidine encapsulated; no wavelength shift was identified for the microspheres containing Cl-amidine, compared with empty microspheres, indicating absence of impurities (Supplementary Figure S1). Cl-amidine absorption spectrum was used to detect its wavelength of maximum absorbance (327 nm) required for further testing using UV-spectrophotometry (Supplementary Figure S2), whilst no further assessment by FTIR was carried out for Cl-amidine alone.

### 2.4. Porosity of Microspheres

All of the P(3HB) microspheres, the empty control, the control with encapsulated rhodamine and with encapsulated Cl-amidine, produced under the same conditions (condition 3 in Table 1) exhibited internal porosity which was measured using the following equation:

ε = (W_2_−W_3_−W_s_)/(W_1_−W_3_).

**Figure 3:**
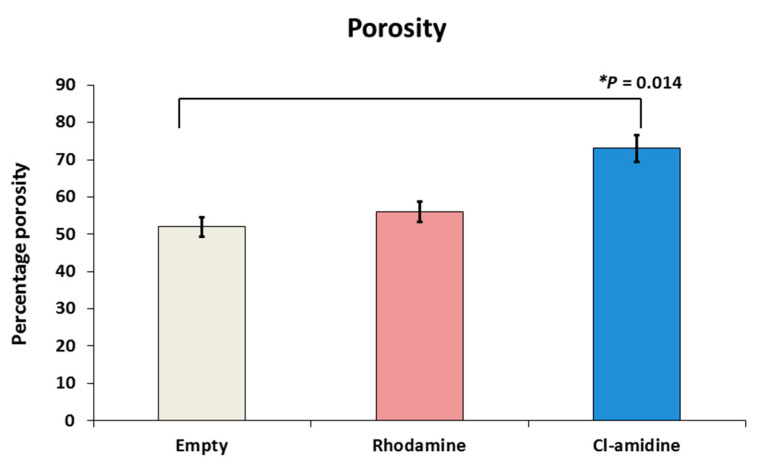
Porosity assessment of P(3HB) microspheres with encapsulated drug. The percentage porosity exhibited by empty, rhodamine and Cl-amidine containing P(3HB) microspheres was 52%, 56% and 73%, respectively; exact *p* values are indicated, and error bars indicate standard deviation (SD); * *p* ≤ 0.05.

The average percentages of porosity for the empty control, rhodamine encapsulated control and Cl-amidine encapsulated P(3HB) microspheres were 52%, 56% and 73%, respectively. This percentage porosity exhibited by the P(3HB) microspheres with encapsulated Cl-amidine was significantly higher than that of the empty control microspheres (*p* = 0.014), whilst there was no significant difference when compared to the control microspheres with rhodamine encapsulated (Figure 3).

### 2.5. Surface Hydrophobicity and Residual Percentage PVA

**Figure 4:**
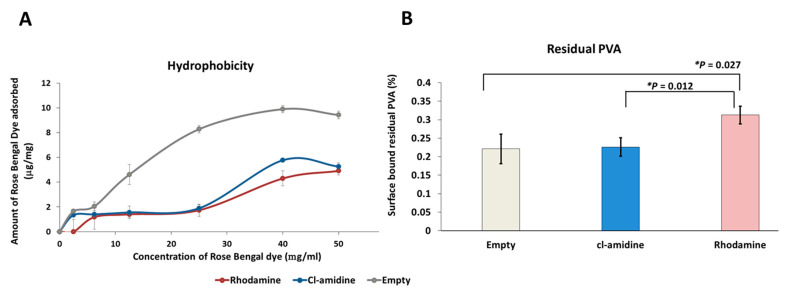
The surface hydrophobicity and residual PVA percentage of the empty, rhodamine and Cl-amidine containing P(3HB) microspheres. (**A**) The hydrophobicity of each type of microspheres according to the amount of surface bound Rose-Bengal dye per each mg of the microsphere sample. (**B**) The residual PVA percentage bound to the surface of each type of microspheres. Exact *p* values are shown; the error bars indicate SD; * *p* ≤ 0.05.

The determination of surface hydrophobicity of the control empty P(3HB) microspheres and those with encapsulated rhodamine and Cl-amidine, produced under condition 3 (Table 1), was carried out by comparing the amount of Rose Bengal dye adsorbed per each mg of the three groups of microspheres. The amount of Rose Bengal dye bound to each mg of the three groups of microspheres increased as the percentage of the surface bound residual PVA decreased (Figure 4A,B). For instance, at the maximum Rose Bengal dye concentration used (50 µg/mL), the empty control P(3HB) microspheres exhibited the highest adsorption by adsorbing 9.42 µg/mg of the dye (Figure 4A) while retaining the lowest percentage of residual PVA on its surface which is 0.221% (Figure 4B). As for the Cl-amidine containing P(3HB) microspheres which adsorbed 5.26 µg/mg (Figure 4A), the microspheres exhibited a percentage of 0.226% of residual PVA (Figure 4B). Finally, the rhodamine encapsulated control microspheres showed the least adsorption of 4.9 µg/mg of the dye (Figure 4A) whilst exhibiting the highest percentage of surface-bound residual PVA that reached 0.312% (Figure 4B).The statistical analysis showed insignificant differences between the empty control and Cl-amidine containing microspheres in terms of the residual percentage PVA. However, comparing the rhodamine containing control microspheres to both, the empty control and microspheres with encapsulated Cl-amidine revealed a significantly higher percentage of residual PVA as indicated by *p* values of 0.027 and 0.012, respectively. Conversely, there were insignificant differences between the three produced batches of microspheres on measuring the surface hydrophobicity upon performing ANOVA analysis (*p* value 0.06). The average amount of dye adsorbed by empty control P(3HB) microspheres, upon applying increasing dye concentrations, was 5.9 µg/mg compared to 2.8 and 2.2 µg/mg for microspheres with encapsulated Cl-amidine and rhodamine, respectively. 

### 2.6. Protein Adsorption Assay (Bradford’s Assay)

**Figure 5:**
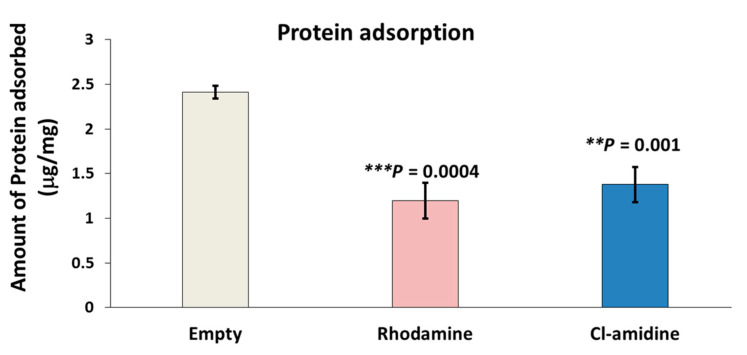
Protein adsorption assay of empty, rhodamine and Cl-amidine P(3HB)-containing microspheres. This figure shows the amount of protein (BSA) adsorbed on the surface of each mg of the three types of microspheres (exact *p* values are indicated, the error bars indicate SD); ** p ≤ 0.01, *** *p* ≤ 0.001.

The empty P(3HB) microspheres exhibited the highest protein adsorption of 2.4 µg/mg of bovine serum albumin (BSA) on its surface which corresponded to retaining 9.42 µg/mg of Rose-Bengal dye (Figure 5). Conversely, control rhodamine containing P(3HB) microspheres adsorbed the least amount of BSA on the surface represented by 1.19 µg/mg while simultaneously possessing the least surface hydrophobicity (4.9 µg/mg) measured using the rose Bengal dye adsorption assay. As for the Cl-amidine containing P(3HB) microspheres, these adsorbed 1.37 µg/mg of BSA which is an intermediate value in between the two other types of microspheres and the same applied to its surface hydrophobicity (5.26 µg/mg) (Figure 5). The protein adsorption values of empty control microspheres were significantly higher by 43% (*p* value = 0.001) and 53% (*p* value = 0.0004) when compared to microspheres with encapsulated Cl-amidine and rhodamine, respectively, whilst no significant difference was seen between Cl-amidine and rhodamine containing spheres.

### 2.7. Cl-Amidine Encapsulation Efficiency and In Vitro Release Kinetics

**Figure 6:**
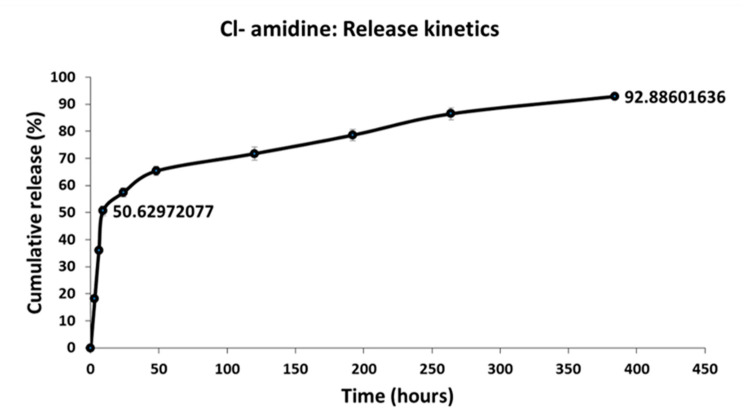
The encapsulation efficiency of Cl-amidine within the P(3HB) microspheres and the release kinetics. This figure shows the cumulative percentage of Cl-amidine released from the P(3HB) microspheres over a period of 16 days.

Cl-amidine release from the P(3HB) microspheres was assessed and expressed as a function of encapsulation efficiency which was found to be 48.10 ± 0.69% for the microspheres. There was a burst release of ~50% in the first 24 h followed by a zero order release from that point up to 16 days, by which point, ~93% of the drug is released (Figure 6).

### 2.8. Cell Culture 

**Figure 7:**
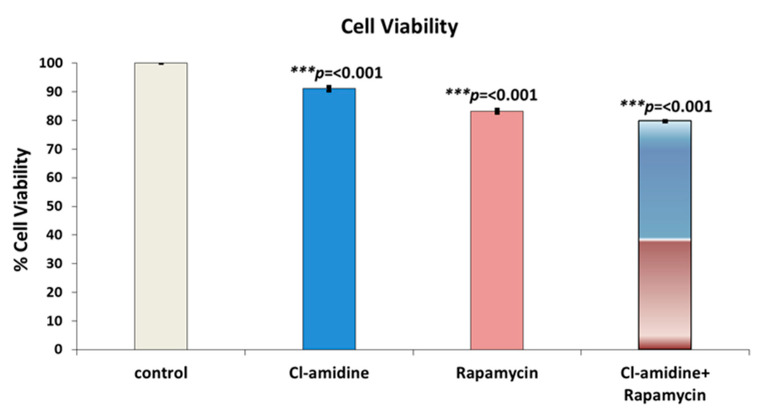
The effect of a combined application of P(3HB) microspheres with encapsulated Cl-amidine along with the anti-proliferative drug (Rapamycin) on the viability of SK-BR-3 breast cancer cells. The bars indicate control (untreated) cells, compared with microspheres with encapsulated Cl-amidine, free rapamycin and Cl-amidine containing spheres in combination with free rapamycin (exact *p* values are indicated, the error bars indicate SD); *** *p* ≤ 0.001.

The mammalian breast cancer cell line SK-BR-3 was treated with the anti-proliferative drug rapamycin, with and without the presence of Cl-amidine encapsulated P(3HB) microspheres. The viability of the breast cancer cell line was found to be 91.1%, 83.2% and 79.7% after being treated with P(3HB) microspheres with encapsulated Cl-amidine, with rapamycin, and with Cl-amidine containing microspheres in combination with rapamycin, respectively (Figure 7). This showed that the combinatorial effect of rapamycin together with the Cl-amidine containing microspheres, on reducing the breast cancer cell line viability, surpassed the effect of the anti-proliferative drug alone by 3.5% (Figure 7). This was further confirmed by the statistical tests which revealed significant differences between the three groups where the combinatorial effect showed *p* values of 2 × 10^−8^ and 1 × 10^−5^ when compared to treatment of the cell line with either Cl-amidine containing microspheres or rapamycin alone, respectively. However, rapamycin application alone exhibited a significant reduction in cell viability compared to the effect of microspheres containing Cl-amidine (*p* value = 4 × 10^−7^).

### 2.9. Vascular Endothelial Growth Factor Expression (VEGF)

**Figure 8:**
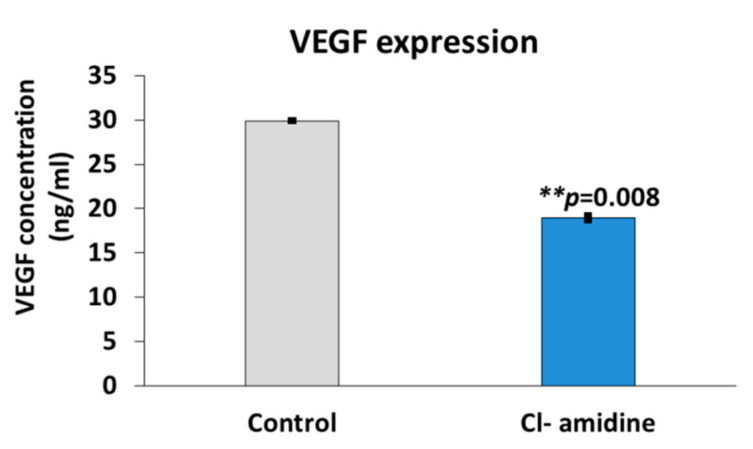
Effects of microspheres with encapsulated Cl-amidine on VEGF expression in breast cancer cells. This figure shows the significant decrease in VEGF expression in SK-BR-3 breast cancer cells following treatment with the P(3HB) microspheres with encapsulated Cl-amidine (exact *p* values are indicated, the error bar indicates SD); ** *p* ≤ 0.01.

The quantification of vascular endothelial growth factor (VEGF) expression using the breast cancer cell line SK-BR-3 was interpreted as an indicator of tumour vascularization and metastasis. The VEGF quantification showed that the cells treated with the P(3HB) microspheres with encapsulated Cl-amidine significantly reduced the expression of VEGF by 36.54% (*p* value = 0.008) when compared with untreated cells (Figure 8).

## 3. Discussion

The current study was aimed at encapsulating the pan-PAD inhibitor Cl-amidine into P(3HB) microspheres for controlled drug delivery, which may be applicable in a range of PAD-related pathologies, including inflammatory, autoimmune and neurodegenerative diseases, as well as cancer. Here, the technique of choice was solid-oil-in-water (s/o/w) emulsion for the preparation of P(3HB) microspheres, which was carried out under three different conditions which varied in the polymer and surfactant concentrations, as well as the stirrer speed, in order to optimize maximum encapsulation efficiency within the microspheres. For microsphere preparation, polyvinyl alcohol (PVA) was used as the emulsifying surfactant to stabilize the double emulsion system through promoting the P(3HB) precipitation in the form of microspheres rather than its precipitation as a polymeric mass as previously observed in the absence of PVA [79,80]. It has been reported in several studies that the varying PVA concentrations can affect the produced microspheres in terms of particle size and encapsulation efficiency. In the current study, the applied concentrations of PVA in both the internal and external emulsion phases were 0.5% *w*/*v* for condition 1 and 1% *w*/*v* for both conditions 2 and 3 (Table 1). This specific PVA range was selected since previously applied PVA concentrations below 0.5% *w*/*v* failed to stabilize the organic phase droplets within the external aqueous phase of the emulsion, leading to their adherence to the magnetic stirrer. Conversely, raising the concentration above 1% *w*/*v* rendered it difficult to obtain the desired size range of the microspheres due to the elevated viscosity of the external emulsion phase, owing to the high molecular weight of PVA [79]. It was thus expected that doubling the PVA concentration from 0.5% *w*/*v* in condition 1 to 1% *w*/*v* in condition 2 for improved emulsion stabilization, whilst retaining the same polymer concentration of 0.013 g per mL of chloroform and stirring speed of 300 rpm for both conditions, would result in the production of microspheres with increased particle size. SEM images of microspheres produced under condition 2 showed a larger diameter range of 50–60 µM (Figure 1A) compared to condition 1 which resulted in a diameter average of 40 µM (Figure 1A). This is explained by the relatively increased viscosity of the external emulsion phase owing to doubling the PVA concentration, thus requiring higher shear force to produce microspheres of a smaller size range [75]. Consequently, to balance this effect, the stirrer speed was increased from 300 rpm in conditions 1 and 2 to 1200 rpm in condition 3 while using the 1% *w*/*v* PVA concentration. Applying this, a significantly smaller size range of the P(3HB) microspheres was produced owing to both the higher shear forces of the increased stirring speed [81,82] and using the higher PVA concentration (1% *w*/*v*) which, together, prevented the coalescence of the double emulsion droplets into larger microparticles [83]. In addition, previous studies [84,85] stated that the polymer concentration in the emulsion phase is directly proportional to the solution viscosity. Thus, decreasing the polymer concentration in this condition (condition 3) to half of its value, from 0.013 g/mL to 0.0065 g/mL, reduced the solution viscosity and consequently facilitated its dispersion into smaller microspheres than those produced under both previous conditions. Furthermore, the PVA concentration used within both the internal and external emulsion phases directly affects the distribution pattern and the encapsulation efficiency of Cl-amidine inside the P(3HB) microspheres. It was reported that increasing the PVA concentration, within both phases, helps to promote the uniform distribution of the encapsulated drug along with improving its encapsulation efficiency. The latter is attributed to the increased availability of PVA in the initial interface between the internal aqueous phase and organic phase as well as covering the second interface between the organic phase and the external aqueous phase which decreases the surface tension significantly and thus prevents leakage of the encapsulated hydrophilic drug from the hydrophobic polymeric system during microspheres production [83,85]. Besides, applying a high concentration of PVA in the internal emulsion phase decelerates the coalescence of internal emulsion droplets; hence, it improves the encapsulation efficiency of the entrapped drug [85]. Accordingly, it is suggested that using the higher PVA concentration of 1% *w*/*v* in condition 3 contributed to achieving a proper encapsulation efficiency of the hydrophilic Cl-amidine within the P(3HB) microspheres. In addition, according to previous studies, the applied concentration of PVA is directly correlated to its residual amount found on the surface of the produced microspheres. Since identifying the amount of residual PVA was carried out for the three types of microspheres produced simultaneously under condition 3, using the same PVA concentration of 1% *w*/*v*; thus, the resulting percentage surface residual PVA among the three species was within a close range of 0.22–0.31% [75]. This range is similar to the results obtained by Zielhuis et al. [86] who reported a range of 0.2–0.3% of residual PVA on PLGA microspheres, whilst representing almost half the values reported by Francis et al. [75], which reached 0.5–0.6% on the surface of P(3HB) microspheres upon applying a PVA concentration of 1% *w*/*v* [75,86]. The presence of residual PVA identified on the microsphere surface is attributed to its molecular arrangement as an amphiphilic surfactant across the organic and aqueous phases of the double emulsion to reduce the surface tension. During the microsphere production, the hydrophobic tail of the PVA binds irreversibly to the polymeric surface within the organic phase upon reaching the critical micelle concentration, whilst retaining its hydrophilic head attached on the microspheres surface despite the final washing step [87,88,89,90]. Consequently, the percentage residual PVA represents its surface-exposed hydrophilic portion, which is inversely proportional to the surface hydrophobicity and protein adsorption properties of the produced P(3HB) microspheres as revealed by this study. According to the current results, at the maximum Rose Bengal dye concentration used (50 µg/mL) to determine surface hydrophobicity, the empty control P(3HB) microspheres exhibited the highest adsorption (Figure 4a) while retaining the lowest percentage of residual PVA on its surface (Figure 4b). Conversely, the control rhodamine encapsulated microspheres showed the least adsorption of the dye (Figure 4a) alongside exhibiting the highest percentage of surface bound residual PVA (Figure 4b) while the microspheres with encapsulated Cl-amidine adsorbed an amount of Rose-Bengal dye which lay in the middle between the other two types of microspheres and the same applied to its percentage of surface bound residual PVA. This inverse relationship of residual PVA and surface hydrophobicity was also confirmed by previous studies [75]. 

As for the protein adsorption, the current study showed that a higher surface hydrophobicity was directly correlated to higher protein (BSA) adsorption onto the surface of the P(3HB) microspheres (Figure 5). This could be explained by the outcomes of a recent study conducted by Fang et al. [91] who applied the Hansen solubility parameters (HSP) as an indicator to identify the polarity of the surface-adsorbed BSA versus the native, unbound BSA [91]. The study revealed proximity between the HSP values of the BSA adsorbed onto polymeric surfaces and those of the hydrophobic Zein protein. In addition, these calculated values proved that the surface-adsorbed BSA is much more hydrophobic compared to the native, unbound BSA. This altered hydrophobic conformation is attributed to BSA denaturation upon polymeric surface adsorption which results in displaying its hydrophobic core, rather than its hydrophilic shell, onto the polymeric surface. Hence, the BSA adsorption onto the P(3HB) microspheres was inversely proportional to their residual PVA content and directly dependent on their surface hydrophobicity [91]. 

Since the main aim of this study was to attempt a controlled release and delivery system for Cl-amidine, the encapsulation efficiency and in vitro release kinetics of Cl-amidine were further assessed. Cl-amidine release from the P(3HB) microspheres was expressed as a function of encapsulation efficiency which was found to be 48.10 ± 0.7% within the microspheres. This value is considered among the maximum encapsulation efficiencies (EE) reported of hydrophilic drugs encapsulated into various polymeric micro/nanospheres. For instance, the EE of the hydrophilic doxycycline in 5002A PLGA microspheres reached a maximum range of (42.12% ± 4.66) to (45% ± 6.31) despite the reported optimization of almost all of the various formulation parameters [92]. Besides, the EE of doxorubicin within poly(3-hydroxybutyrate-co-3-hydroxyvalerate) nanoparticles was 22.9% ± 1.7 [93], whilst encapsulating vancomycin within the same polymeric microspheres achieved an EE of 27.3% [94]. A slightly higher value of vancomycin EE was reported in case of using poly(lactide-co-glycolide)– methoxypoly(ethylene glycol) microspheres which is equal to 55.2% [95]. Additionally, loading of alendronate sodium into polycaprolactone based nanoparticles achieved an EE of 34% [96], whilst P(3HB) microspheres, similar to those applied in this study, with encapsulated gentamicin exhibited a similar EE of 48% [75]. However, an outlier to these reported values was the EE of novel oligo (3-hydroxybutyrate-graft-hyaluronic acid) Oligo (3HB-g-HA) microspheres with encapsulated gentamicin, which reached around 71.3 ± 2.5 % [97]. Thus, among the future perspectives of this research study would be the improvement of EE of the hydrophilic Cl-amidine through testing a number of mechanisms reported by recent literature which include pH-adjustment of the external aqueous phase of the emulsion system and applying a small-scale liquid–liquid partitioning to provide the maximum concentration of Cl-amidine free-base (unionized form) in the organic polymeric phase [98]. 

Following the EE, a pivotal element of the polymeric drug delivery systems was addressed which is porosity. Porosity was the key determinant of Cl-amidine diffusivity and release from within the P(3HB) microspheres [99]. Due to the hydrophilic nature of Cl-amidine (70 mg/mL solubility in water according to Selleckchem and Merck indices), its molecules tend to form a honeycomb porous structure across certain areas within the microsphere matrices which contain the formed water channels upon solvent evaporation during microsphere production, where Cl-amidine is efficiently distributed, encapsulated and further released through accelerating the water uptake and thus penetration into the encapsulating microspheres [75,95]. This honeycomb porous structure is formed while applying the double emulsion–solvent evaporation technique for the production of the P(3HB) microspheres with hydrophilic Cl-amidine as well as the hydrophilic rhodamine encapsulated control [39,100]. Initially, the aqueous phase containing either dissolved Cl-amidine or rhodamine is dispersed into an organic phase of the P(3HB) polymer, dissolved into chloroform, forming the first water-in-oil (w_1_/o) emulsion. Then, this (w_1_/o) emulsion is also dispersed into a second aqueous phase containing the hydrophilic, emulsifying surfactant, PVA, to finally form the double emulsion system (w_1_/o/w_2_). This system then undergoes solvent evaporation of the chloroform. During chloroform evaporation, the P(3HB) polymer shrinks towards the core of the emulsion forming microdroplets which constitute the honeycomb porous structure that contributes to the increased system porosity compared to the empty control P(3HB) microspheres produced using a single emulsion technique. Besides, this specific structure is reported to be optimal for efficient encapsulation and release of the highly hydrophilic drugs. In addition, the outermost layer of the precipitating P(3HB) polymer also forms holes through which either the encapsulated Cl-amidine or rhodamine could be partially released. Consequently, this double emulsion-solvent evaporation technique has been repeatedly used in several studies to improve the entrapment and encapsulation efficiency of various hydrophilic drugs [85,101]. 

Another mechanism of Cl-amidine release was through the biodegradation process of the P(3HB) microspheres. P(3HB) biodegradation takes place through surface erosion due to the crystallinity and hydrophobicity of the P(3HB) polymeric backbone [102]. This hydrophobicity contributes to the relatively slow rate of water penetration into the polymeric matrix. Consequently, the vast majority of the microspheres biodegradation process occurs at the surface in the form of layer-by-layer hydrolysis, a process which could be described as the hydrolytic peeling of the polymeric microspheres from the surface inwards which is accompanied by the release of the encapsulated drugs entrapped within each layer, until the polymeric matrix experiences mass loss [103,104,105]. Thus, this mechanism of surface erosion is a desirable element of the polymeric drug delivery system since it contributes to achieving a stable, zero-order pattern of sustained drug release which maintains the release of a constant amount of the encapsulated drug from within the microspheres, per unit time, regardless of the initial drug concentration [106,107]. According to the current study results, there was a burst release of ~50% of Cl-amidine in the first 24 h attributed to the surface associated drug [95]. This was then followed by a slower phase of zero order release from that point up to 16 days, by which point approximately 93% of the drug had been released (Figure 6).

This indicated that the Cl-amidine encapsulated within the P(3HB) microspheres can be used as a new delivery system for targeted Cl-amidine release, at controlled rates, over longer periods of time, ranging from days to weeks. Consequently, this could provide novel application approaches for PAD inhibition in autoimmune diseases, cancer, as well as in neurological conditions, where Cl-amidine has previously been shown to be neuroprotective in CNS injury [6,7]. As deimination is associated with a range of neurodegenerative pathologies including AD, MS and PD [3,19,22,23,26], the prospect of controlled longer-term delivery of PAD inhibitors, including Cl-amidine may offer novel treatment approaches.

In addition to the established neuroprotective roles of Cl-amidine, its anticancer effects have also been investigated in several studies. For instance, Cl-amidine acts as an effective modulator of extracellular vesicle release and cargo content from cancer cells, also enhancing the efficiency of chemotherapeutic agents such as 5-fluorouracil (5-FU) and TMZ in prostate, breast and brain cancer [3,8,33,34]. Furthermore, Cl-amidine contributes to cancer cell apoptosis through induction of p53 nuclear accumulation, which in turn, causes overexpression of the OKL38 gene, an apoptosis mediator [36,46]. 

In our current study, the mammalian breast cancer cell line SK-BR-3 was treated with the anti-proliferative drug rapamycin with and without the presence of P(3HB) microspheres with encapsulated Cl-amidine. The effect of combining rapamycin with microspheres with encapsulated Cl-amidine showed a 3.5% reduction in breast cancer cell viability in a 24 h period compared with cells treated with rapamycin only (Figure 7). Thus, Cl-amidine released from the microspheres could effectively sensitise cancer cells to chemotherapy and this may offer novel approaches for the use of controlled delivery of Cl-amidine in cancer. Furthermore, an effect of P(3HB) microspheres with encapsulated Cl-amidine on tumour vascularization and metastasis was assessed as a function of VEGF expression. SK-BR-3 cells treated with Cl-amidine containing P(3HB) microspheres were indeed found to have a significantly reduced expression of VEGF (by 36.54%), when compared with untreated cells (Figure 8). Future work will aim at assessing the effectivity of the P(3HB) microspheres with encapsulated Cl-amidine in vivo, alongside further optimization for encapsulation efficiency and application in various disease models. This type of controlled delivery system may also be applicable for other PAD inhibitors, including isozyme-specific PAD inhibitors, with potentially tailored application for different diseases.

## 4. Materials and Methods

### 4.1. Materials

P(3HB) was produced according to previously established methods [43] and was isolated using the soxhlet extraction method from the Gram-positive bacteria *Bacillus subtilis* OK2 strain which was previously provided as a gift from Prof Fujio Kawamura, Department of Life Sciences, Rikkyo University, Japan. All the chemicals used for the growth of *Bacillus subtilis* OK2 strain, and the polymer extraction, were purchased from Sigma-Aldrich Co (Gillingham, UK) and VWR Chemicals (Poole, UK). The kits and reagents used for characterization of the microspheres were purchased from Bio-Rad Laboratories, Inc. (Watford, UK), and Thermo Fisher Scientific (Hemel Hempstead, UK). The PAD inhibitor Cl-amidine (Calbiochem, 506282) was obtained from Merck (Darmstadt, Germany).

### 4.2. Production of P(3HB) Microspheres with Encapsulated Rhodamine and Cl-Amidine 

Synthesis of P(3HB) microspheres was carried out using a solid-in-oil-in-water emulsion technique. Different P(3HB) solutions in chloroform were prepared by dissolving 0.13 g of the polymer in either 10 or 20 mL of chloroform, in airtight bottles, overnight to ensure complete dissolution. Then, the polymer solution was homogenized for duration of 5 min using a homogenizer mixer. This was then transferred, in a drop-wise manner, to 40 mL of either 0.5% or 1% *w*/*v* of aqueous polyvinyl alcohol (PVA) solution, stirred at 600 rpm, to form the aqueous first solid-in-oil emulsion. In case of drug encapsulated microspheres, this PVA solution would contain 2 mg of either dissolved rhodamine or Cl-amidine. P(3HB) microspheres with encapsulated rhodamine were produced as a control due to its hydrophilic nature, similar to Cl-amidine, and thus the physical characteristics of the microspheres could be compared [76,77]. In addition, rhodamine is a fluorescent dye which would allow imaging of the P(3HB) microsphere localization upon in-vivo testing using confocal microscopy (future work). Finally, the formed emulsion would be vortexed for 10 min and homogenized for 15 min using a homogenizer mixer before its dispersion into an external aqueous phase of 200 mL PVA solution. Hence, the second oil-in-water emulsion was formed which was then stirred at either 300 or 1200 rpm for 4 h to allow evaporation of the chloroform, used as the solvent. Consequently, the empty P(3HB) microspheres with encapsulated rhodamine/Cl-amidine were produced and centrifuged at a speed of 4600 rpm (g = 2370) for 20 min. The supernatant was then discarded before the microspheres were washed with water and then subjected to a second centrifugation cycle, frozen at 4 °C and finally freeze dried (Savant Modulyo D Freeze drier, Thermoelectron Corp, Cheshire, UK). This double emulsion method was applied since both the encapsulated compound (Cl-amidine) and control (rhodamine) drugs are hydrophilic [39,75,100,108].

### 4.3. Characterization of P(3HB) Microspheres with and without Encapsulated Rhodamine or Cl-Amidine

#### 4.3.1. Surface Morphology and Microstructure Characterization 

A JOEL 5610LV scanning electron microscope (SEM) was used to carry out the micro-structural as well as the surface morphology studies on the samples of microspheres. Aluminium stubs with 8 mm diameter were used on which the samples were placed to get coated with gold by EMITECH-K550, a gold spluttering device. The operating pressure and deposition current of 7 × 10^−2^ bar and 20 mA, respectively, were used for 2 min. Then, to prevent the polymer incineration by the beam heat, the maximum acceleration voltage of 15 kV was applied while taking the SEM images (Carried out at UCL Eastman Dental Institute, London, UK).

#### 4.3.2. Porosity

Liquid displacement method was used for measuring the microsphere porosity (ε) [109]. The weight of a measuring cylinder was determined before and after adding 5 mL of methanol. To this point, a specific weight of microspheres was added to this methanol followed by using the vortex for 5 min to enhance the methanol penetration into all of the pore spaces of the microspheres. Removal of the excess methanol which exceeded the 5 mL mark was carried out before weighing the cylinder again. The final step was the recovery of the microspheres from the methanol and recording the weight of the methanol along with the cylinder. Finally, the porosity was calculated according to the following formula:

ε = (W_2_ − W_3_ − W_s_)/(W_1_ − W_3_), where

W_1_ represents the weight of the cylinder filled with 5 mL of methanol before addition of the microsphere sample, W_2_ represents the weight of the cylinder together with methanol and the microsphere sample, after removal of the amount of methanol which exceeded the 5 mL mark, W_3_ represents the weight of the cylinder filled with methanol after removal of the microsphere sample penetrated by methanol and W_s_ is the specific weight of the microspheres sample added to the methanol [75].

#### 4.3.3. Determination of Residual PVA content

Quantification of the residual polyvinyl alcohol depends on the ability of iodine molecule to form a coloured complex between its adjacent hydroxyl groups [110]. This quantification was carried out using 2 mg of empty control, Cl-amidine and control rhodamine containing P(3HB) microspheres which were incubated with 2 mL of 0.5 M sodium hydroxide (NaOH) in a water bath adjusted at 60 °C for 15 min. This was then neutralized using 900 µL of 1 N hydrochloric acid (HCL). Finally, distilled water was added to adjust the volume to 5 mL followed by the addition of 3 mL of 0.65 M solution of boric acid, 0.5 mL of iodine solution (I2)/Potassium iodide (KI) (0.05 M/0.15 M) and 1.5 mL of distilled water to each type of the microsphere samples. Finally, this complex was incubated at room temperature for 15 min and its absorbance was measured at 690 nm using the Novaspec II Visible spectrophotometer (Fisher Scientific, Loughborough, UK). In addition, a standard graph of PVA was developed under exactly the same conditions [75].

#### 4.3.4. Determination of Surface Hydrophobicity

Empty P(3HB) microspheres (2 mg) with encapsulated Cl-amidine and control rhodamine samples were incubated at room temperature with varying concentrations of Rose Bengal dye, ranging from 0–50 µg/mL for a duration of 3 h. Controls were prepared using dye solutions of the same concentrations used with the microspheres. Following incubation, the samples of microspheres were centrifuged using a microcentrifuge (Sorvall legend RT, Fisher Scientific, Loughborough, UK) at a speed of 12,000 rpm (*g* = 8064) for 10 min duration. The absorbance of the unbound dye in the supernatant was measured at 542.7 nm using the Novaspec II Visible spectrophotometer. Finally, the amount of Rose Bengal dye adsorbed on the surface of the microspheres was determined using the prepared controls as references.

#### 4.3.5. Protein Adsorption Studies Using Bradford’s Assay

P(3HB) microspheres (2 mg), empty control, rhodamine control and with encapsulated Cl-amidine samples were immersed in a BSA solution of 25 µg/mL concentration. Then, the samples were incubated at 37 °C for 24 h. Simultaneously, standard dilutions of BSA were treated under the same conditions to obtain the calibration curve. The samples were centrifuged at 12,000 rpm (*g* = 8064). The Bradford reagent (Bio-Rad Laboratories, Watford, UK) was added to the supernatant to carry out Bradford’s assay which was used to determine the amount of unbound BSA. Briefly, 1 mL of Bradford’s reagent was added to the supernatant of diluted samples and standards followed by incubation at room temperature for 25 min. Finally, absorbance was measured at 280 nm to determine the concentration of BSA in the supernatant such that the protein adsorbed on the surface of the samples could be calculated using the following equation

Q = (C_i_ − C_f_) V/m.

where C_i_ is the initial concentration of BSA, C_f_ is the final BSA concentration in the supernatant after centrifugation while V is the total volume of the solution and m is the weight of the samples.

### 4.4. In Vitro Drug Release Kinetics 

The in vitro drug release kinetics were assessed over a period of 16 days using an incubator with temperature maintained at 37 °C. P(3HB) microsphere samples which had been encapsulated with 2 mg of Cl-amidine, were immersed in 2 mL of phosphate buffer saline (PBS) prepared at pH of 7.4, used to mimic the blood plasma and body fluids due to its isotonicity, and kept at 37 °C. Microsphere samples of 1 mL were drawn periodically in triplicates and fresh PBS was added. Cl-amidine content of the samples was analysed using UV spectrometry at 327 nm using the Lambda 35 UV/VIS Spectrometer, Perkin Elmer, Beaconsfield, UK, and its concentration quantified using a standard curve.

### 4.5. Drug Quantification through Encapsulation Efficiency Calculation

The percentage drug encapsulation efficiency was calculated using the following formula:

%EE = Experimental drug loading/Theoretical drug loading.

To determine the EE values, 10 mg of the Cl-amidine encapsulated P(3HB) microspheres were dissolved in 1 mL of chloroform, then vortexed for 10 min. In the case of the hydrophilic drug Cl-amidine, 2 mL of Phosphate Buffer Saline (PBS) was added, and the mixture was vortexed for further 10 min. This mixture was then centrifuged at 4600 rpm for 10 min to separate out the hydrophilic Cl-amidine in the water phase where it was quantified by UV spectrometry at 327 nm using the Lambda 35 UV/VIS Spectrometer, PerkinElmer, Beaconsfield, UK.

### 4.6. Cell Culture 

SK-BR-3 cells [111] were grown in DMEM media, supplemented with 10% foetal calf serum, and 1% *w*/*v* penicillin and 1% *w*/*v* streptomycin solution, as described by ATCC [112]. The medium was changed every 2–3 days. The cell cultures were maintained at 37 °C, 5% CO_2_ and passaged based on 60–70% confluence, by trypsin treatment for 10 min. Following cell detachment, fresh medium was added to the cell suspension, which was then centrifuged at 1000 rpm for 10 min. The resulting cell pellet was suspended in fresh medium and transferred to 75 cm^2^ tissue culture flasks. Cells were seeded in 24-well plates, with a density of 5 × 10^5^/mL and incubated for 24 h to allow attachment. 

Triplicates of SK-BR-3 cells were treated either with 1 mg/mL each of P(3HB) microspheres with encapsulated Cl-amidine, with 5 ng/mL of rapamycin, or with a combination of both 5 ng/mL of rapamycin and 1 mg/mL of microspheres with encapsulated Cl-amidine. These were incubated for 24 h, whereas untreated SK-BR-3 cells were used as a positive control, and cell viability was assessed using MTT assay [113]. 

Briefly, 100 μL of MTT reagent was added to each sample and incubated for 4 h. The culture media was removed and 100 μL of DMSO was added and incubated for a further 2 h. The absorbance was read at 590 nm.

### 4.7. Effects on VEGF Expression 

VEGF expression within the control (untreated) SK-BR-3 cells and those treated with P(3HB) microspheres with encapsulated Cl-amidine was calculated using the VEGF human ELISA kit, following the manufacturer’s protocol (Thermo Fisher Scientific, UK).The samples and controls were added to ELISA plates with immobilized antibody. The VEGF antibody complex (Thermo Fisher Scientific, Hemel Hempstead, UK) was washed using the washing buffer to remove any unbound samples and then treated with a second detector antibody with a conjugate enzyme that binds to a different epitope on the target antibody. The microplates were then washed to remove any unbound substances and a substrate specific to the enzyme, was added, forming a coloured complex. The absorbance of this complex was read at 540 nm.

### 4.8. Statistical Analysis

All experiments were carried out in triplicate, and data was expressed accompanied by their mean standard deviation (SD). Student’s *t* test, ANOVA and Bonferroni correction tests were used to compare the data and to analyse significant differences among the assessed factors. The significant differences were represented as * *p* < 0.05, ** *p* < 0.01 and *** *p* < 0.001.

## 5. Conclusions

The current study developed a new drug delivery method for the pan-PAD inhibitor Cl-amidine by encapsulation into Poly(3-hydroxybutyrate) microspheres. Cl-amidine encapsulation in P(3HB) microspheres was optimized, and the release kinetics of Cl-amidine from the microspheres were assessed in vitro and expressed as a function of encapsulation efficiency. A burst release of ~50% Cl-amidine was observed in the first 24 h, followed by a zero-order release from that point up to 16 days, at which time point ~93% of the drug had been released. SK-BR-3 breast cancer cells treated with Cl-amidine encapsulated microspheres were found to have 36.5% reduction in VEGF expression when compared with untreated SK-BR-3 cells, indicative of anti-cancerous effects via controlled release of Cl-amidine from P(3HB) microspheres. Cl-amidine delivery via P(3HB) microspheres may provide both controlled and prolonged release delivery of this PAD inhibitor in cancer as well as in a range of other PAD-related diseases, including neurodegenerative and autoimmune pathologies. Future studies will need to assess the reported P(3HB) microspheres with encapsulated Cl-amidine in further in vitro and in vivo models, as well as testing this controlled drug delivery system for other PAD inhibitors.

## Figures and Tables

**Table 1:** Process optimization of empty control, Cl-amidine and control rhodamine encapsulated P(3HB) microspheres.

Condition	Drug	Drug Loading (mg)	P(3HB) Concentration (g/mL)	PVA Concentration (%*w*/*v*)	Stirrer Rate (rpm)
1	Cl-amidine	2	0.013	0.5%	300
2	Cl-amidine	2	0.013	1%	300
3	Cl-amidine	2	0.0065	1%	1200
3	Rhodamine Control	2	0.0065	1%	1200
3	None/Control	-	0.0065	1%	1200

## Data Availability

All data for this study are included within the manuscript and Supplementary Data.

## Supplementary Materials

The following are available online at https://www.mdpi.com/article/10.3390/ijms222312852/s1.
