# Supplementary material for: Controlled Delivery of Pan-PAD-Inhibitor Cl-Amidine Using Poly(3-Hydroxybutyrate) Microspheres
Source: Int J Mol Sci. 2021 Nov 27;22(23):12852. doi: 10.3390/ijms222312852 (PMC8658019; doi:10.3390/ijms222312852)
Supplement: Supplementary file 1 [file ijms-22-12852-s001.zip › ijms-1468173-supplementary.pdf]

Supplementary Figure S1

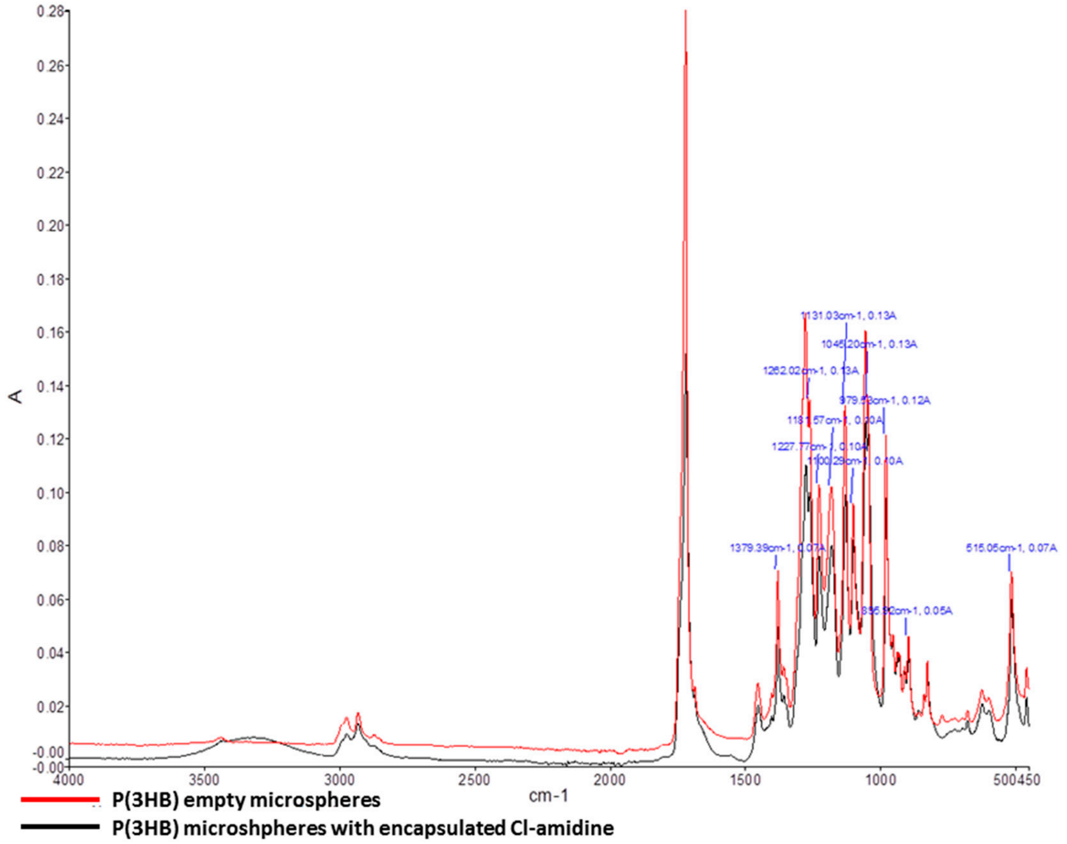

Figure S1. Overlay FTIR of empty and Cl-amidine encapsulated P(3HB) microspheres. The red line represents empty P(3HB) microspheres and the black line represents P(3HB) microspheres with encapsulated Cl-amidine.

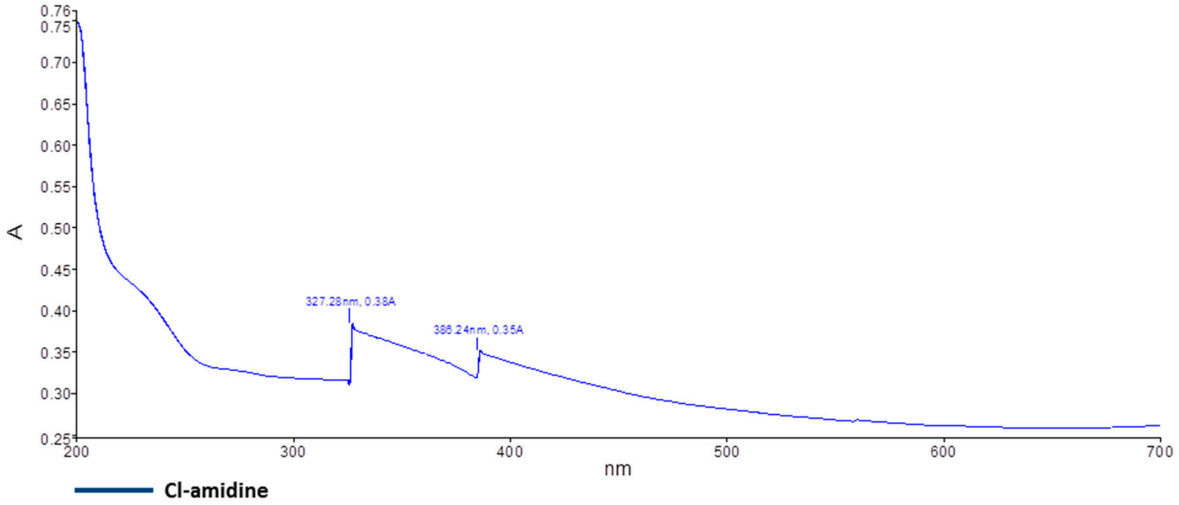

Figure S2. Cl-amidine absorption spectrum was used to detect its wave-length of maximum absorbance (327 nm) required for further testing using UV-spectrophotometry.
